# Enhancing natriuretic peptide bioactivity prevents bleomycin-induced pulmonary fibrosis

**DOI:** 10.1186/1471-2210-11-S1-P3

**Published:** 2011-08-01

**Authors:** Reshma S Baliga, Sarah L Trinder, Christopher J Scotton, Rachel C Chambers, Raymond J MacAllister, Adrian J Hobbs

**Affiliations:** 1 Department of Pharmacology, University College London, Gower Street, London WC1E 6BT, UK; 2 Centre for Respiratory Research, University College London, Gower Street, London WC1E 6BT, UK; 3 Centre for Clinical Pharmacology, University College London, Gower Street, London WC1E 6BT, UK

## Background

Idiopathic pulmonary fibrosis (IPF) is a progressive fibro-proliferative disorder of unknown etiology, with no effective treatment regimen, and a median survival of just 3–5 years following diagnosis [1]. Pulmonary Hypertension (PH) often complicates IPF and is associated with higher mortality. Augmentation of endogenous natriuretic peptide (NP) stimulated cGMP, using a neutral endopeptidase inhibitor (NEPi) in combination with a phosphodiesterase 5 inhibitor (PDE5i), synergistically prevents pathogenesis in a hypoxic model of PH [2]. Herein, we assessed the efficacy of this novel NEPi/PDE5i combination therapy in a model of pulmonary fibrosis.

## Methods

Littermate wild-type (WT) and NP receptor-A knockout (NPR-A KO) mice (male; 20-25g; C57BLK6 background) were instilled with saline (control) or Bleomycin (Bleo; 1mg/kg) in the absence or presence of the PDE5i sildenafil (30mg/kg/day; p.o.) or the NEPi ecadotril (60mg/kg/day; p.o.). Haemodynamic and morphological parameters were measured 14 days post Bleo administration.

## Results

**Figure 1:**
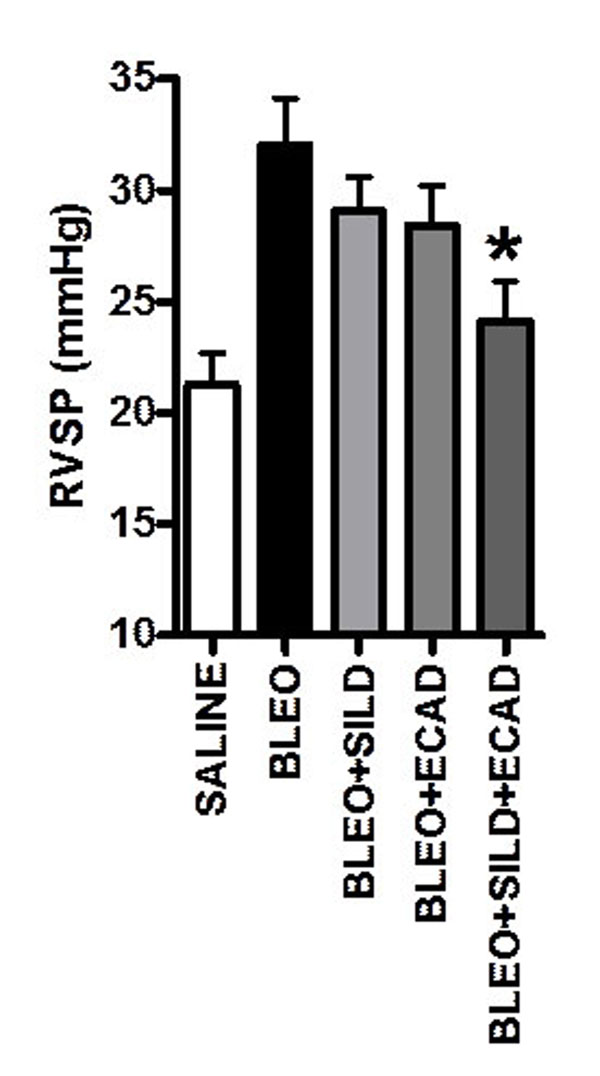
Right ventricular systolic pressure (RVSP) in saline-treated mice (n=14), and Bleo-treated (n=8) animals in the absence and presence of sildenafil (SILD, n=7), ecadotril (ECAD; n=8) and combination treatment (n=9). *P<0.05 versus Bleo alone.

Bleo treatment caused a significant rise in right ventricular systolic pressure (RSVP) in WT mice that was not reduced by either sildenafil or ecadotril alone, but significantly inhibited by the combination (Figure 1). Bleo administration also resulted in right ventricular hypertrophy (RVH); this was prevented by treatment with sildenafil, ecadotril or the combination (RV/LV+S; Control: 0.2486±0.005, Bleo: 0.3054±0.0177, Bleo+sildenafil: 0.258±0.006*, Bleo+ecadotril: 0.261±0.008*, Bleo+combination: 0.247±0.008*; *P<0.05 versus Bleo).

Bleo instillation enhanced total lung collagen accumulation (index of fibrosis) which was ameliorated by concomitant administration of sildenafil, ecadotril or the combination (Control: 1.692±0.2, Bleo: 4.4±0.35, Bleo+combination: 2.124±0.12*; *P<0.05 versus Bleo).

NPR-A KO mice, lacking the bioactivity of ANP and BNP, exhibited an exaggerated haemodynamic response to Bleo (WT Bleo RSVP: 31.77±1.96mmHg, KO Bleo RSVP: 41.68±2.78mmHg; P<0.05), and the beneficial effects of the combination therapy were lost (KO Bleo+combination RSVP: 39.84±2.41mmHg; *P*>0.05 versus KO Bleo). A similar loss in efficacy of the combination treatment was observed against RVH (KO Bleo RV/LV+S: 0.252±0.008, KO Bleo+combination RV/LV+S: 0.234±0.017; *P*>0.05). Figure 1.

## Conclusion

The NEPi/PDE5i combination is effective in ameliorating both the haemodynamic aberrations and fibrotic lung disease in this model. This beneficial pharmacodynamic activity depends on the bioactivity of NPs, which represent an endogenous protective mechanism limiting disease progression. This work substantiates the therapeutic potential of manipulating NP and cyclic GMP signalling for PH and IPF.
